# Corrigendum: WCoP Abstracts

**DOI:** 10.1002/psp4.12595

**Published:** 2021-02-01

*CPT:Pharmacometrics and Systems Pharmacology*, 9: S1–28. https://doi.org/10.1002/psp4.12497

The published version of this supplement was missing one abstract. It follows below:

POPULATION PHARMACOKINETICS OF VANCOMYCIN IN PAKISTANI PATIENTS. **M. Usman**, M. Muaaz Munir and H. Rasheed; University of Veterinary and Animal Sciences, Lahore, Pakistan

**BACKGROUND:** Despite of more than 200 million diverse population of Pakistan, the area of population pharmacokinetic modeling is still neglected and there is a need of popPK studies, particularly for narrow therapeutic index drugs including anti‐microbial agents such as vancomycin.

**METHODS:** Population pharmacokinetic analysis was performed on NONMEM by using data of 162 samples obtained from 30 patients at different time intervals after administration of vancomycin standard dose. One‐compartment model was applied as the base model with first‐order conditional estimation method with interaction (FOCE‐I). The influence of different covariates was investigated by stepwise covariate modeling and the final model was evaluated by goodness of fit plots and bootstrap analysis using 1000 datasets.

**RESULTS:** In the final model, the mean values (RSE%) for vancomycin clearance (CL) and volume of distribution (VD) were 2.76 L/h (2%) and 21.7 L (3.3%) respectively. The inter‐individual variability for vancomycin CL was 37.9 %. The IOV was observed by proportional error which was 0.185. Vancomycin CL was significantly influenced by creatinine CL (CLCR) in a stepwise covariate modeling. This influence can be quantified by equation CLj = CLref × (1 + 0.0059 * (CRCL − 115.55)).

**CONCLUSION:** Vancomycin CL in Pakistani patients is comparable with other populations and is influenced by CRCL. Therefore, dose in Pakistani patients should be decided on the basis of CRCL of the patients in order to ensure the safe and effective use of vancomycin.

